# Enhancing panic disorder treatment with mobile-aided case management: an exploratory study based on a 3-year cohort analysis

**DOI:** 10.3389/fpsyt.2023.1203194

**Published:** 2023-10-19

**Authors:** Chan-hen Tsai, Mesakh Christian, Feipei Lai

**Affiliations:** ^1^Graduate Institute of Biomedical Electronics and Bioinformatics, National Taiwan University, Taipei, Taiwan; ^2^Department of Psychiatry, En Chu Kong Hospital, New Taipei City, Taiwan; ^3^Department of Computer Science and Information Engineering, National Taiwan University, Taipei, Taiwan

**Keywords:** case management, panic disorder, panic attack, quality of care, mobile-aided intervention, maintenance treatment

## Abstract

**Background:**

Individuals with panic disorder frequently face ongoing symptoms, suboptimal treatment adherence, and increased relapse rates. Although mobile health interventions have shown promise in improving treatment outcomes for numerous mental health conditions, their effectiveness, specifically for panic disorder, has yet to be determined.

**Objective:**

This study investigates the effects of a mobile-aided case management program on symptom reduction and quality of care among individuals with panic disorder.

**Methods:**

This 3-year cohort study enrolled 138 participants diagnosed with panic disorder. One hundred and eight participants joined the mobile-aided case management group and 30 in the treatment-as-usual group. Data were collected at baseline, 3-month, 6-month, and 12-month treatment checkpoints using self-report questionnaires, in-depth interviews, direct observation, and medical record analysis.

**Results:**

During the maintenance treatment phase, the mobile-assisted case management group decreased both panic severity (*p* = 0.008) and state anxiety (*p* = 0.016) more than the control group at 6 months. Participants who underwent case management experienced enhanced control over panic symptoms, heightened self-awareness, and elevated interpersonal support.

**Conclusion:**

The mobile-aided case management is beneficial in managing panic disorder, especially maintenance treatment.

## 1. Introduction

Panic disorder is a common anxiety disorder marked by recurring, unexpected panic attacks, persistent worry or apprehension about having subsequent panic attacks, and causing substantial distress and functional disruption (1). Panic attack frequently involves numerous physical symptoms like chest pain, difficulty breathing, palpitations, sweating, trembling, the feeling of choking, nausea, dizziness, chills or heat sensations, numbness, feelings of unreality, or being detached from oneself; and psychological symptoms such as fear of losing control, going crazy, or fear of dying. The complex nature of these presentations can sometimes lead to confusion for cross-disciplinary clinicians. We hypothesized case managers could enhance communication with multidisciplinary clinicians. They can also track patients over time, thus simplifying symptom control and adherence to psychiatric management. Additionally, mobile-supported technology could aid case managers in observing and monitoring patients’ status outside clinics. This study sought to determine whether mobile-supported case management can improve treatment outcomes in various domains, including symptom relief, reduce emergency visits, or promote treatment adherence.

Case management is a collaborative approach to coordinating and providing care for individuals with complex needs. It involves assessing, planning, implementing, and evaluating care plans and resources to ensure optimal health outcomes (2, 3). Case management has been demonstrated to enhance the quality of care for individuals with severe mental illnesses (4, 5), including schizophrenia, severe depression, and substance use disorder (6). Case management’s central focus is strengthening engagement, assessment, personal planning, and resource acquisition (4). Compared to standard care, intensive case management may reduce hospitalization, increase retention, and improve social functioning for patients with severe mental illnesses (5). Case management facilitates personalized care, involves a coordinated approach to the treatment plan, identifies barriers to treatment, and implements flexible adjustments to care plans beyond clinical settings. It also provides support during crises, such as hospitalizations or mental health emergencies, by coordinating resources and managing the situation for individuals and their families. However, few studies (7–10) examine the effectiveness of case management specifically in treating panic disorder at the time of our research.

The treatment-as-usual (TAU) approach for panic disorder typically consists of pharmacotherapy (11, 12) and psychological and/or behavioral interventions, such as cognitive-behavioral therapy (CBT) (13–15) or relaxation. Extensive evidence supports the effectiveness of these TAU approaches in reducing the frequency and intensity of panic attacks and improving the overall quality of life (16). However, several unmet needs persist (17–19). These include more accessible and scalable treatment options, greater personalization and adaptability of interventions, and improved long-term outcomes. Mobile-aided case management offers potential solutions to these challenges by enhancing the accessibility of treatment, allowing for real-time tracking and personalized support, and promoting sustained engagement and self-management (20).

Patients with panic disorder sometimes struggle to consistently follow their prescribed treatment plan, resulting in insufficient adherence and impacting overall effectiveness. In previous studies, the unremitted rate of panic disorder is around 42–46% (21, 22), and subthreshold symptoms persist even after 12-week treatment (23). Treatment-resistant panic disorder (24) is associated with higher severity, longer duration, co-occurring mental and physical conditions, early onset, elevated anxiety sensitivity, and avoidant behavior. It can sometimes worsen due to various psychosocial stressors, including life events, chronic stress, limited social support, and childhood adversity. These stressors may result in sub-threshold panic symptoms, such as restricted panic attacks or phobic avoidance. Guided self-help (25) is sometimes effective in relieving these stressors. Implementing case management in panic disorder may facilitate access to appropriate treatment, such as medication, psychological or behavioral treatment, guided self-help, and navigating the multidisciplinary healthcare system.

In recent years, mobile-aided and internet-based interventions have emerged as promising tools for case managers in evaluating patients with panic disorder. Mobile-aided interventions involve mobile devices like smartphone apps (26, 27) for anxiety disorders such as generalized anxiety disorder (28) and tablets to collect data and provide patient feedback. For example, patients can use mobile apps to track their symptoms and receive personalized feedback from their case managers. Internet-based interventions involve using online platforms to support and treat patients. These interventions can be accessed from anywhere with an internet connection, making them particularly useful for patients who live in remote or underserved areas (29). Growing evidence supports the internet-based approach for anxiety and depressive disorder. For example, researchers found equivalent reduced psychiatric and somatic symptoms both in face-to-face and internet-based cognitive behavioral therapy (30, 31). Furthermore, internet-based interventions can significantly reduce healthcare utilization and costs (32, 33) and provide therapists or case managers with valuable information about their patient’s symptoms and progress, allowing them to make more informed treatment decisions (34, 35). These interventions can also improve communication between patients and case managers, as patients can easily share information and ask questions through digital platforms. Prior research (36, 37) has reviewed the efficacy of smartphone apps on anxiety and depressive disorders and showed some benefits. Depressive symptoms were significantly reduced from smartphone apps than control conditions (*g* = 0.38, 95% CI: 0.24–0.52, *p* < 0.001) (37). However, for apps in preadolescents and adolescents, only two small randomized trials and one case study failed to demonstrate a significant effect of three apps on intended mental health outcomes (36).

We hypothesized case management brings less recurrent rate or better recovery in anxiety and depressive disorder. In this report, we sought to demonstrate the efficacy of combining mobile-aided service with case management for panic disorder.

## 2. Methods

The design of this entire study is initially a prospective cohort study and development plan for the panic prediction model (38). However, we aim to evaluate our implemented system’s clinical efficacy and benefit in the presented article. It is an exploratory study conducted by aggregating data from part of a cohort analysis and comparing if mobile-aided case management outperforms treatment as usual. Please see Sections 2.1–2.4 for the process detail.

### 2.1. Participants

This study enrolled 108 participants from psychiatric clinics at a community hospital between June 15, 2020, and March 22, 2023, to receive mobile-aided case management (MCM) augmented with regular care. The eligibility criteria included being an adult aged 18 or older and having a Diagnostic and Statistical Manual of Mental Disorders, Fifth Edition (DSM-5) (1) diagnosis of panic disorder, either with or without agoraphobia. Additionally, we matched a subset of 30 patients diagnosed with panic disorder but had undergone treatment as usual (TAU). The treatment checkpoints period is 1 year for each enrolled participant. It is standardized for all participants, and no difference between groups. However, most participants kept maintenance treatment after 1 year and followed up in the same hospital after the study to avoid the recurrence of panic disorder.

Certified psychiatrists conducted the diagnostic interview with standardized procedure by DSM-5 and recorded in electronic medical records. Trained psychiatric researchers further applied MINI [Mini-International Neuropsychiatric Interview (39), a short structured diagnostic interview] to screen all psychiatric comorbidities. Panic disorder is the primary diagnosis for all cases. All MINI diagnoses were checked (such as OCD, PTSD, bipolar disorder, substance use disorder … etc.). However, comorbidities other than GAD and depression are relatively rare and are not discussed in this paper.

### 2.2. Panic monitoring system

**Figure 1:**
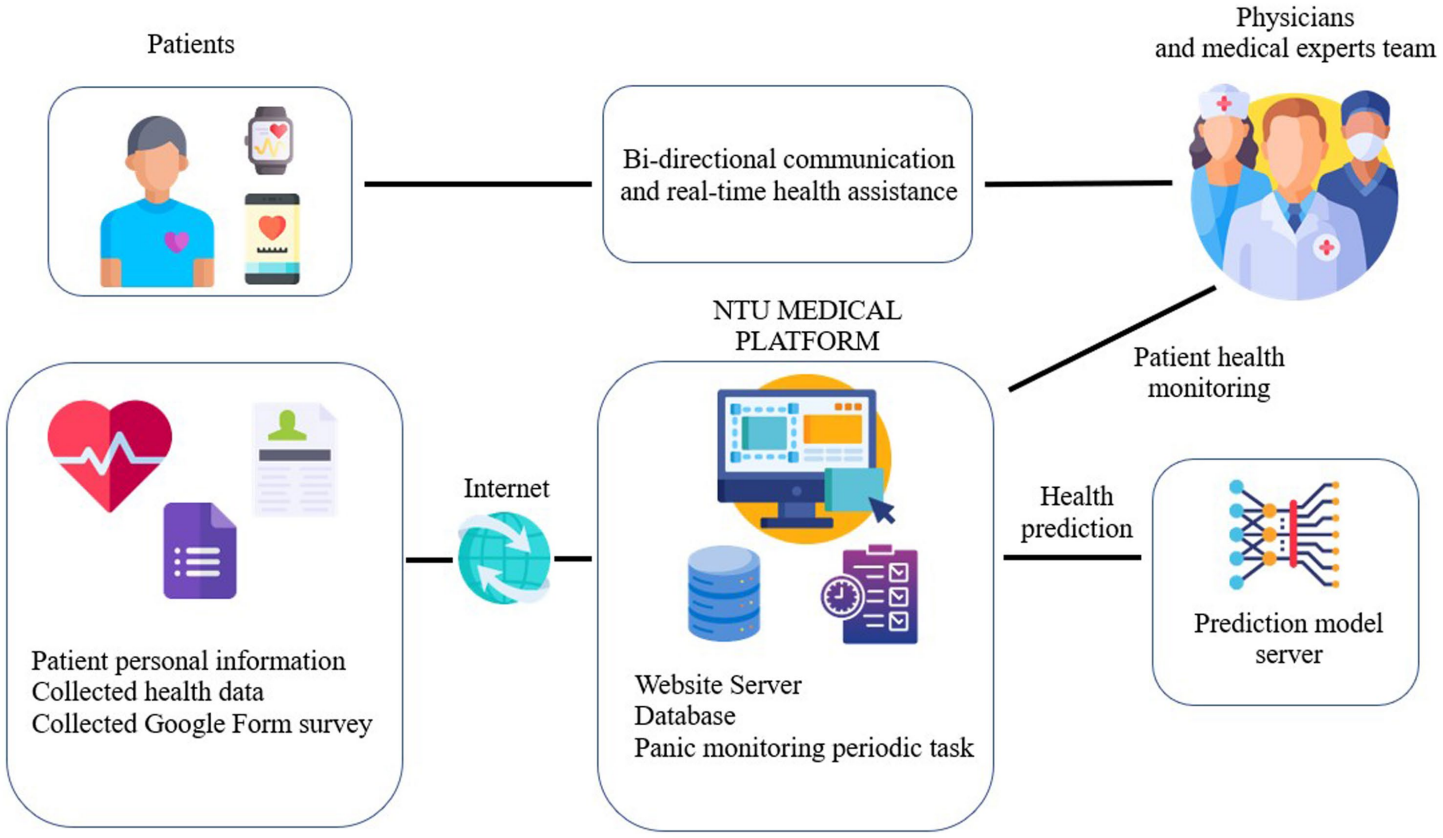
A high-level overview of the relationship between patients, the medical team, and the panic monitoring system.

This study recruited three case managers with nursing expertise. Our team built the management system to deliver individualized and timely treatment for panic disorder patients outside clinics, as illustrated in Figure 1. The design includes three parts: (1) A machine learning-based panic attack prediction model (38) embedded on the National Taiwan University (NTU) Medical Platform server, which connects three cloud servers, including PostagreSQL, influxDB, and Google Sheets. Supplementary Part 1 presents the information structure. (2) The mobile application served as a platform for patients to track their symptoms, access personalized resources, and communicate with their case managers. Please see Supplementary Parts 2, 3. The application also allowed patients to set goals and engage in self-guided stress management to reinforce the skills learned during in-person therapy sessions. (3) Patients in the TAU-MCM group completed the PDSS (Panic Disorder Severity Scale) (40, 41), STAI-S (State-Trait Anxiety Inventory-State subscale), and STAI-T (State-Trait Anxiety Inventory-Trait subscale) (42, 43) questionnaires on the mobile application weekly to evaluate participants’ panic severity, state anxiety, and trait anxiety. And they reported real-time panic attack symptoms through the application.

Our team designed a panic monitoring system using the machine learning model (38) within a monolithic service architecture. This unified system comprises message workers that activate the NTU platform for regular data transmission, a backend Application Programming Interface (API) for processing incoming requests, and a dedicated model server for deploying our model. As a result, we primarily obtain patient data and provide predictive outcomes from the model. The case manager reviewed these data every week. If the average PDSS score exceeded ten or patients experienced more than two panic attacks per week, the case manager would directly contact patients on the telephone and notify the referring psychiatrists.

### 2.3. Mobile-aided case management vs. treatment as usual

This study employed two distinct treatment approaches for patients diagnosed with panic disorder: Treatment as Usual (TAU) and Mobile-aided Case Management (MCM) with TAU. The aim was to compare the efficacy of these methods in managing panic disorder symptoms. We invited all participants to the MCM-TAU group initially. Those who did not want contact with a case manager, but agreed to regular surveys and clinic follow-ups, formed the TAU group. Due to the limited number of available case managers, participants unable to access this resource also joined the TAU group. To ensure various experiences and detect meaningful differences, we strategically increased participant numbers in the MCM + TAU group.

1. Treatment as Usual (TAU): TAU refers to the standard care provided to patients with panic disorder, typically including a combination of pharmacotherapy and cognitive behavioral therapy. The pharmacological treatment includes escitalopram, sertraline, and venlafaxine as first-line treatment and low dosage augmented use of benzodiazepines.
2. Mobile-aided Case Management (MCM) with TAU: MCM involves integrating mobile technology, such as smartphones and wearable devices, to supplement TAU. This approach provides additional support and patient monitoring through mobile applications and remote contact with healthcare professionals. In this study, MCM was implemented alongside TAU, with patients receiving pharmacotherapy, CBT, and mobile-aided case management services. The MCM component consisted of regular contact with case managers via telephone and a dedicated mobile application. The contact frequency in the MCM group was determined based on each patient’s needs and symptom severity, with a minimum of one contact per week. This contact aimed to monitor the patient’s progress, provide support, and address any concerns or questions about their treatment plan. The wearable devices are smartwatches that collect patients’ daily physical activities, sleep, and heart rate during the study period; we presented the wearable outcome in the previously published article (38). The current article will focus on symptom reduction and the potential benefit of mobile-aided case management. The case managers monitored only questionnaire data, so we did not include wearables’ information here.

In treating panic disorder, psychiatrists guide patients through specific self-guided CBT techniques. They help patients understand the disorder’s nature, suggest tracking panic episodes using journals or apps, and teach deep breathing techniques for symptom relief. Exposure therapy is introduced by gradually facing trigger situations. Patients learn to challenge cognitive distortions and remain calm during anxiety peaks. Additionally, psychiatrists recommend having an emergency plan, like deep breathing or contacting someone during sudden attacks.

In summary, this study sought to compare the efficacy of TAU and MCM with TAU in managing panic disorder symptoms. Both approaches included pharmacotherapy and self-guided CBT, while the MCM group received additional support through mobile technology and remote case management services.

### 2.4. Assessment

We investigated the effects between groups using a mixed-methods (quantitative and qualitative) approach. For both groups, quantitatively, we examined changes in panic disorder severity [measured by PDSS (40, 41)], state anxiety [evaluated using STAI-S (42, 43)], and trait anxiety (assessed by STAI-T) at baseline, 3-month, and 6-month time points post-enrollment. We evaluated general anxiety and depressive indicators using the BDI [Beck Depression Inventory-II (44)] and BAI [Beck Anxiety Inventory (45)] at the time of registration, as well as at the 3-month and 6-month treatment checkpoints intervals. Given that the primary emphasis of the study was on changes in panic severity, we conducted an additional PDSS assessment at the 12-month juncture. We applied in-depth interviews, medical records analysis, and direct observation for the qualitative component.

Case managers assist individuals with panic disorder to stay committed to their treatment plan, ensure they have access to essential services, and support them in preventing relapses. In the MCM group, the contact frequency varies based on each patient’s needs and symptom severity, but at least one contact per week via apps or telephone. This regular contact aims to oversee the patient’s progress, provide support, and address any treatment-related concerns or questions. Additional contacts are tailored based on the case manager’s understanding of the patient. Typically, an extra contact becomes necessary if the weekly PDSS Question One score exceeds 1, indicating that a recurrent panic attack occurred in the previous week, or if the weekly PDSS sum score (ranging from 0–28) surpasses 10, indicating a slight illness. However, the frequency of additional contacts is a joint decision between the case manager and the patient, factoring in the patient’s needs and personal situation. At times, increased life stress or a depressed mood might also necessitate more frequent contact.

The practical interventions from case managers include: (1) If a case’s PDSS sum exceeds 10 or PDSS Question One exceeds 1 in the previous week, case managers contact patients using mobile apps or phones to ascertain recent panic attack details: frequency, intensity, and specific somatic symptoms. They can also monitor these symptoms through the app interface presented in Supplementary Part 2. (2) For patients with an existing self-guided CBT plan; case managers assess the present triggers, responses to these triggers, and self-help actions such as relaxation, anxiolytic use, attention diversion, and their efficacy in reducing panic attacks. (3) Case managers evaluate recent medication compliance in patients, effects or side effects experienced from the medications, and the patient’s comprehension and confidence in ongoing treatments. (4) They probe into any emergency visits stemming from panic attacks (which is not ideal) or any unplanned self-help strategies, aiming to bolster patients’ self-management during panic episodes. (5) During the insight assessment, case managers inquire if patients discern whether their discomfort originates from physiological or psychological sources. They also explore the patients’ approaches to handling this discomfort and anticipated outcomes. (6) Lastly, case managers notify patients about their upcoming psychiatric appointments.

The anticipated impact on patients includes: (1) Interactions with case managers offer patients interpersonal support, a crucial factor in mental health management. (2) These interactions make patients more aware of their condition and the dynamics of a panic attack. As a result, they prioritize psychiatric care over frequenting varied internal medicine clinics or emergency departments in search of physiological causes. (3) In terms of medication, patients recognize the significance of drugs, commonly selective serotonin-reuptake inhibitors, and opt for sustained use. (4) Patients learn to relax effectively. Skillful relaxation, before or during a panic attack, or proactive stress management (like ensuring less demanding work schedules for those with stress-induced panic attacks) diminishes the likelihood and severity of subsequent attacks.

The participants for the qualitative interviews were selected using a purposive sampling technique. This approach allowed for picking individuals who had completed at least three-month treatments and could provide rich, detailed, and relevant information about their treatment experiences and the support they received. We conducted a semi-structured interview with a guide (46); please see Supplementary Part 4 for the detailed interview questions. We interviewed 36 participants from the MCM-TAU group and ten from the TAU group for 80 min per person. The main topic of the thematic analysis included patient satisfaction, adherence to treatment plans, health status or behavior changes, access to resources, and care coordination. We also reviewed any emergency visits due to panic attacks for medical record analysis. The observers include three case managers, four referred psychiatrists, and nurses from the clinics. Two trained researchers conducted the qualitative interviews in a clinical research setting. They had no prior relationship with the participants to reduce potential biases. Each interview was conducted privately, ensuring confidentiality and comfort for the participants.

### 2.5. Statistical analysis

We compared the before-after self-control difference score of PDSS, STAI-S, STAI-T, BDI, and BAI (five outcome variables) between the MCM-TAU and TAU groups. We did not use the crossectional mean score of both groups because each participant’s initial severity differed at enrollment. Later, we employed a *z*-test with the numpy (47) and scipy (48) modules in Python 3.0 environment to compare the two groups; choosing the significance level (α) to a more stringent value of 0.001 to reduce the potential likelihood of a Type I error from a non-randomized group assignment. The covariates were: gender, age, and if comorbid with generalized anxiety disorder, depression, and agoraphobia. We applied Repeated Measures Analysis of Covariance (RMANCOVA) analysis by R language 4.3.0 for each outcome variable separately for the TAU and MCM+ TAU groups. It calculated adjusted values for the outcome variables considering the effects of the multiple covariates. Please see Supplementary Part 5 for detail. The comparison involved multiple time points analysis. We listed these results in Table 1. Finally, we calculated and compared the non-adjusted and adjusted means, 95% confidence intervals of the difference, and Bonferroni corrected *p*-values between the groups.

**Table 1:** Comparison of TAU and MCM + TAU for panic disorder.

	TAU	MCM + TAU	95% confidence interval of the difference	*p*-value
**1. Demography factors**				
Number of participants	*n* = 30	*n* = 108		
Average age (SD)	45.1 (12.8)	47.2 (15.5)	[−6.57, 3.57]	0.561
Female to male ratio	1.3 (17/13)	1.4 (63/45)	[−0.19. 0.22]	0.873
Comorbid with agoraphobia (%)	*n* = 6 (20%)	*n* = 18 (16.7%)	[−0.10, 0.22]	0.457
Comorbid with GAD (%)	*n* = 13 (43.3%)	*n* = 30 (27.8%)	[−0.02, 0.38]	0.077
Comorbid with depression (%)	*n* = 7 (23.3%)	*n* = 16 (14.8%)	[0.01, 0.33]	0.048
**2. Panic disorder severity change**				
PDSS at baseline (SD)	10.4 (3.2)	8.1 (5.1)	[0.34, 4.58]	0.003
Adjusted PDSS at baseline (SD)	11.5 (1.0)	9.5 (0.8)	[−3.94, 0.00]	0.051
PDSS change at 3 months (SD)	−5.5 (2.8)	−6.2 (2.5)	[−0.42, 1.43]	0.260
Adjusted PDSS change at 3 months (SD)	−4.9 (0.8)	−5.3 (0.7)	[−1.23, 0.60]	0.494
PDSS change at 6 months (SD)	−6.0 (2.1)	−7.8 (3.1)	[0.85, 2.77]	0.003
Adjusted PDSS change at 6 months (SD)	−5.9 (1.5)	−7.5 (2.2)	[−2.90, −0.45]	0.008*
PDSS change at 12 months (SD)	−5.2 (2.1)	−6.4 (2.5)	[0.29, 2.07]	0.020
Adjusted PDSS change at 12 months (SD)	−5.5 (0.9)	−6.6 (1.1)	[−2.15, −0.08]	0.035*
**3. State anxiety change**				
STAI-S at baseline (SD)	46.1 (7.7)	46.0 (10.3)	[−4.24, 4.82]	0.890
Adjusted STAI-S at baseline (SD)	45.5 (2.0)	45.0 (1.7)	[−4.62, 3.62]	0.811
STAI-S change at 3 months (SD)	−9.5 (2.4)	−11.6 (5.2)	[0.80, 3.29]	0.036
Adjusted STAI-S change at 3 months (SD)	−9.7 (1.7)	−12.0 (1.4)	[−4.24, −0.25]	0.028*
STAI-S change at 6 months (SD)	−15.5 (2.5)	−17.0 (3.6)	[0.37, 2.69]	0.029
Adjusted STAI-S change at 6 months (SD)	−15.6 (1.3)	−17.4 (0.7)	[−3.19, −0.34]	0.016*
**4. Trait anxiety change**				
STAI-T at baseline (SD)	43.1 (6.3)	46.5 (8.3)	[−6.84, 0.78]	0.020
Adjusted STAI-T at baseline (SD)	42.6 (1.7)	46.1 (1.4)	[0.10, 6.87]	0.044*
STAI-T change at 3 months (SD)	−12.1 (2.8)	−11.3 (3.5)	[−2.04, 0.36]	0.224
Adjusted STAI-T change at 3 months (SD)	−12.5 (1.4)	−11.8 (0.7)	[−0.71, 2.12]	0.325
STAI-T change at 6 months (SD)	−8.6 (3.6)	−8.1 (4.1)	[−2.23, 1.14]	0.522
Adjusted STAI-T change at 6 months (SD)	−8.0 (0.7)	−7.2 (1.0)	[−0.99, 2.47]	0.398
**5. General anxiety change**				
BAI at baseline (SD)	19.2 (5.5)	21.1 (13.0)	[−7.06, 3.00]	0.248
Adjusted BAI at baseline (SD)	21.0 (2.4)	23.6 (2.0)	[−2.29, 7.56]	0.291
BAI change at 3 months (SD)	−6.9 (3.4)	−9.0 (2.6)	[0.74, 3.41]	<0.001
Adjusted BAI change at 3 months (SD)	−6.6 (1.8)	−8.9 (2.7)	[−3.43, −1.1]	<0.001*
BAI change at 6 months (SD)	−4.7 (3.0)	−12.4 (4.1)	[6.33, 9.03]	<0.001
Adjusted BAI change at 6 months (SD)	−4.8 (7.2)	−12.8 (8.1)	[−9.58, −6.27]	<0.001*
**6. Depression change**				
BDI at baseline (SD)	18.0 (8.2)	14.0 (10.8)	[−0.29, 8.80]	0.031
Adjusted BDI at baseline (SD)	19.7 (2.1)	16.5 (1.8)	[−7.62, 1.07]	0.138
BDI change at 3 months (SD)	−2.0 (0.8)	−3.1 (2.5)	[0.65, 1.73]	0.013
Adjusted BDI change at 3 months (SD)	−2.0 (0.5)	−3.0 (0.4)	[−2.04, −0.13]	0.027*
BDI change at 6 months (SD)	−3.4 (1.0)	−4.2 (2.0)	[0.28, 1.28]	0.036
Adjusted BDI change at 6 months (SD)	−4.2 (0.9)	−5.4 (0.9)	[−1.84, −0.45]	0.001*

*Bonferroni-adjusted difference < 0.05 reach significance.

SD, standard deviation; GAD, generalized anxiety disorder; PDSS, Panic Disorder Severity Scale; STAI-S, State–Trait Anxiety Inventory-State subscale; STAI-T, State–Trait Anxiety Inventory-Trait subscale; TAU, Treatment as Usual; MCM, Mobile-aided Case Management.

For qualitative data, our team used thematic analysis for in-depth interviews, direct observation, and medical chart reviews with Python Natural Language Toolkit (NLTK) (49) library, version 3.6.2. and NVivo software (50), version 12. Relevant quotes, descriptions, or examples from comments were identified and analyzed. Due to the small sample size, dichotomous outcomes, like remission status and emergency department visits, were analyzed using Fisher’s exact tests. The qualitative data from the interviews were transcribed verbatim and analyzed using thematic analysis. This process involved multiple readings of the interview transcripts, generating initial codes, grouping these into potential themes, reviewing and refining these themes to ensure they accurately represented the interview data, and using these themes to gain insights into patients’ experiences with the treatment and the support they received.

## 3. Results

### 3.1. Demography and symptom improvement

Table 1 enumerates participants and their mean age, sex distribution, and prevalent psychiatric comorbidities, including agoraphobia, generalized anxiety disorder, and depression. We also recorded the PDSS, STAI-S, STAI-T, BDI, and BAI sum score changes here. Significant differences (*p* < 0.05) exist in covariate-adjusted PDSS change at six and 12 months, STAI-S, BAI, and BDI change at 3 and 6 months.

Given the disparity in sample sizes (*n* = 108 vs. *n* = 30), we used Levene’s Test (51) to ensure consistent variances across groups and mitigate potential statistical bias. The results showed constant variances for gender [*F* (1, 136) = 0.026, *p* = 0.871], age [*F* (1, 136) = 3.467, *p* = 0.065], GAD [*F* (1, 136) = 3.886, *p* = 0.051], and AG [*F* (1, 136) = 0.673, *p* = 0.413]. Only depression displayed a variance concern [*F* (1, 136) = 7.612, *p* = 0.007]. Nonetheless, these findings support the use of RMANCOVA. The relevant code was provided in the modified Supplementary Part 7.

The differences of particular interest were those with statistically significant adjusted *p*-values. Please see Table 1 (2, 6) for detail.

For PDSS at 6 months, the mean improvement for MCM + TAU was −7.5 (SD: 2.2) compared to TAU’s mean improvement of 5.9 (SD: 1.5), resulting in a difference of 1.6 (*p* = 0.008, CI: −2.09 to −0.45). At 12 months, MCM + TAU’s mean improvement was 6.6 (SD: 1.1) which is better than TAU’s mean improvement of 5.5 (SD: 0.9), and the difference between them was 1.1 (*p* = 0.035, CI: −2.15 to −0.08).

For STAI-S at 3 months, MCM + TAU showed a mean improvement of −12.0 (SD: 1.4) compared to TAU’s mean improvement of −9.7 (SD: 1.7), a difference of 2.3 (*p* = 0.028, CI: −4.24 to −0.25). At 6 months, the mean improvement for MCM + TAU was −17.4 (SD: 0.7) whereas TAU’s was −15.6 (SD: 1.3), yielding a difference of 1.8 (*p* = 0.016, CI: −3.19 to −0.34).

For BAI (General anxiety change) at 3 months, MCM + TAU improved by a mean of −8.9 (SD: 2.7), while TAU’s mean improvement was −6.6 (SD: 1.8), with a significant difference of 2.3 (*p* < 0.001, CI: −3.43 to −1.1). At 6 months, the difference widened with MCM + TAU’s mean improvement of −12.8 (SD: 8.1) compared to TAU’s mean improvement of −4.8 (SD: 7.2), a staggering difference of 8.0 (*p* < 0.001, CI: −9.58 to −6.27).

Finally, for BDI at 3 months, the mean improvement for MCM + TAU was −3.0 (SD: 0.4) against TAU’s − 2.0 (SD: 0.5), a difference of 1.0 (*p* = 0.027, CI: −2.04 to −0.13). At 6 months, the mean difference increased slightly with MCM + TAU improving by −5.4 (SD: 0.9) while TAU improved by −4.2 (SD: 0.9), resulting in a difference of 1.2 (*p* = 0.001, CI: −1.84 to −0.45). However, Levene’s Test for depression at baseline indicates a variance concern [*F* (1, 136) = 7.612, *p* = 0.0066] that could suggest bias. Thus, this improvement should be interpreted with caution.

### 3.2. Qualitative result

Given the appropriate methodologies for smaller sample sizes, distinct patterns emerge from the MCM + TAU and TAU groups. We demonstrated the results of remission status and emergency department visits at 3 months by Fisher’s exact test in Table 2. The status of remission in our interview refers to a period lasting at least 3 months, during which an individual does not experience a panic attack or significant worry about having one. A higher percentage of patients (8 out of 10) reported complete remission in the TAU group than the MCM + TAU group (13 out of 36); however, no significant difference between the groups (odds ratio = 0.141, value of *p* = 0.028). For emergency department visits, none have been to the emergency department due to panic attacks in the MCM + TAU group; However, 3 of 10 participants in the TAU group visited the emergency once due to panic attacks. They acknowledged their emergency visit because they were unsure if this was a panic symptom, especially chest pain, and difficulty breathing.

**Table 2:** Fisher’s exact test in qualitative assessment at 3 months.

	Full remission	No remission	Total
**1. Subjective panic disorder remission from in-depth interviews**			
MCM + TAU	13	23	36
TAU	8	2	10
**2. Emergency visit due to panic attacks**			
	**Emergency visit**	**No visit**	**Total**
MCM + TAU	0	36	36
TAU	3	7	10

Fisher’s exact test: Odds Ratio = 0.141, *p-*value = 0.028.

Fisher’s exact test: Odds Ratio: 0.0, *p*-value: 0.008.

A thematic analysis was conducted using the Python NLTK library and NVivo on the qualitative data from semi-structured interviews. This process helped identify frequently occurring words and phrases, allowing us to pinpoint critical themes and sentiments in the participants’ responses. The identified themes from thematic analysis are interpersonal support, reassurance, self-recognition, care coordination, and medical adherence. From in-depth interviews, 18 of the 36 participants from the MCM + TAU group reported high satisfaction with interpersonal support from the case managers through face-to-face or telephone interviews. Twenty participants reported they could manage panic symptoms and felt reassured and improved self-recognition with regular case management. Both groups were satisfied with care coordination. However, the MCM + TAU group reported more access to medical professionals and increased motivation to adhere to treatment plans because of an increased understanding of the panic disorder. Twenty-one of the thirty participants said they were motivated to keep the maintenance phase of treatment despite being remitted from panic symptoms.

Each theme was supported by substantial evidence from participant responses. For instance, the theme of Interpersonal support was evident in a response such as: “The support from my case manager has been instrumental in my recovery. Our regular phone check-ins make me feel like I am not alone in dealing with this.” Similarly, the theme of reassurance emerged from comments like: “Having regular case management sessions made me feel more secure about handling my panic symptoms.” The process also illuminated a strong presence of Self-recognition in statements like: “With the treatment, I’ve become more aware of what triggers my panic attacks and how to manage them effectively.” Care coordination was echoed in sentiments like: “The coordination between my case manager and the doctor has been seamless. I’ve been able to get the help I need when I need it.” Lastly, Medical adherence responses such as: “Understanding my disorder and the purpose behind each part of the treatment plan has motivated me to adhere to it.”

## 4. Discussion

### 4.1. Principal finding

In summary, case management is a practical approach to treating panic disorder, and mobile-aided interventions can be helpful tools for case managers in remotely evaluating patients. Patients with mobile-aided case management show significant improvement in panic disorder severity (*p* = 0.008) and state anxiety (*p* = 0.016) over TAU at 6 months. The effect for panic disorder severity remains significant at 12 months (*p* = 0.035). They also felt reassurance and interpersonal support, more understanding of panic disorders, less emergency need, and higher satisfaction with the care. However, the two groups have no significant difference in trait anxiety change at 3 and 6 months.

In Table 1 (5, 6), the assessment of general anxiety, conducted using the BAI, exhibited a substantial decrease in the MCM + TAU group compared to the TAU group at 3- and 6-month marks. This result aligns with our findings regarding state anxiety. We observed a similar trend with depression, as measured by the BDI. There was an improvement in depression, despite both groups initially having only mild (ranging from 14–19) BDI scores.

Our research studies found that case management can effectively reduce panic disorder severity, state anxiety, and general anxiety during the maintenance phase of treatment at six-month compared with TAU. It helps individuals with panic disorder remain engaged with their treatment plan, ensures access to necessary services, and supports preventing relapse. However, the efficacy of case management for panic disorder severity during the 3-month acute treatment phase was not significantly different from that of the TAU group. We propose that individuals in both groups, during the acute phase, may have received comparable pharmacotherapy and frequent psychiatrist consultations, which helped maintain medication compliance and symptom management. The trait anxiety change does not differ between groups at 3 and 6 months, suggesting that mobile-aided case management alone may not add on benefit in treating trait anxiety.

We discovered that the mobile-aided approach enhances case managers’ evaluation efficiency compared to conventional case management. The system facilitated communication through mobile applications, a benefit supported by themes of care coordination derived from comprehensive interviews. Regularly monitoring questionnaires submitted via the platform allowed case managers to focus on individuals exhibiting severe recent symptoms, enabling them to offer prompt interpersonal support and re-education, potentially decreasing the likelihood of emergency visits. However, it’s important to note that our control group for this study was TAU, not conventional case management. As such, the benefits observed require further validation.

### 4.2. Strength

This study is valuable in combining mobiles-apps and smartwatches with the case management system and emphasizes panic disorder. The observation period is 3 years and provides quantitative and qualitative evidence of mobile-aided case management’s effect on treating panic disorder. We add our clinical insight to growing proof (52), demonstrating that adding app-supported mobile monitoring can improve outcomes for anxiety and depressive disorders.

In summary, integrating case management with mobile monitoring technologies offers a promising avenue for enhancing the treatment of panic disorder. Our research suggests that these approaches can lead to improved outcomes compared with TAU alone, addressing some unmet needs in managing panic disorder.

### 4.3. Limitation

The two groups were non-randomized assigned because it was initially an observational cohort study and the purpose of developing a panic attack prediction system (38). Thus, we must be aware of selection bias that the TAU group is prone to less adherence to treatment. Secondly, we should consider the learning effects, recall bias from questionnaires, and measurement errors from the before-after self-control measurement. Our in-depth interview used purposive sampling and could only approach 46 participants. These may lead to limited generalizability and biased selection from a population of more cooperative patients. All the cases were from a single community hospital; further external testing is needed.

### 4.4. Recommendations and implications

Implementing case management is associated with cost-effective analysis and potential changes in public-health policies; we plan to conduct a cross-disciplinary study to evaluate if mobile-aided case management improves the quality of care and reduces medical expenses. Demographic factors like education level, job type, and family status might influence the application of mobile-aided case management. Future research designs should take these factors into account.

## 5. Conclusion

Mobile-aided case management for panic disorder demonstrates benefits over treatment as usual (TAU) in several domains: significant panic and state-anxiety symptoms reduction in the maintenance treatment phase, self-recognition of symptom control, increased adherence to medication, and reduced emergency visits due to panic attacks. Further robust studies should be conducted for confirmation.

## Data availability statement

The datasets presented in this article are not readily available because the dataset contains identifiable patients’ private information. Requests to access the datasets should be directed to C-hT, tsaichanhen@gmail.com.

## Ethics statement

The studies involving humans were approved by En Chu Kong Hospital research plan (ECKH D11101). The studies were conducted in accordance with the local legislation and institutional requirements. The participants provided their written informed consent to participate in this study.

## Author contributions

C-hT designed the study and completed the data analysis and writing. MC supported the data analysis and information engineering. FL directed and supervised the project. All authors contributed to the article and approved the submitted version.
