# Supplementary material for: Enhancing panic disorder treatment with mobile-aided case management: an exploratory study based on a 3-year cohort analysis
Source: Front Psychiatry. 2023 Oct 19;14:1203194. doi: 10.3389/fpsyt.2023.1203194 (PMC10620526; doi:10.3389/fpsyt.2023.1203194)
Supplement: Supplementary file 1 [file Data_Sheet_1.docx]

Supplementary Materials

Part 1 Architecture of Mobile-assisted Panic Monitoring System
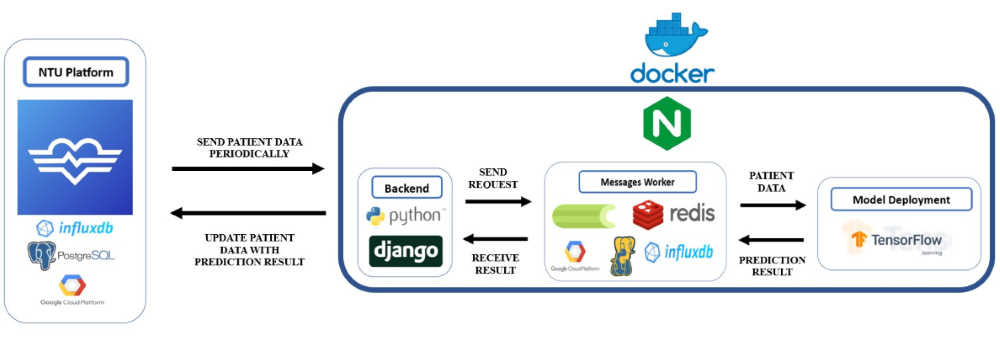


NTU platform will send patient data to our model server (the right box) periodically, then receive the monitoring result back to the platform. Our unified system includes message workers to activate the NTU platform for data transmission, a backend Application Programming Interface (API) to process requests and a dedicated model server for deploying our model. This design allows us to obtain patient data and provide predictive outcomes. We encapsulate the model within a monolithic service design for the machine learning prediction system. Scaling can be achieved by deploying multiple system replicas as the user base grows.

Part 2 Smartphone App: A user interface that collects psychological questionnaires, panic attack symptoms, and basic profile


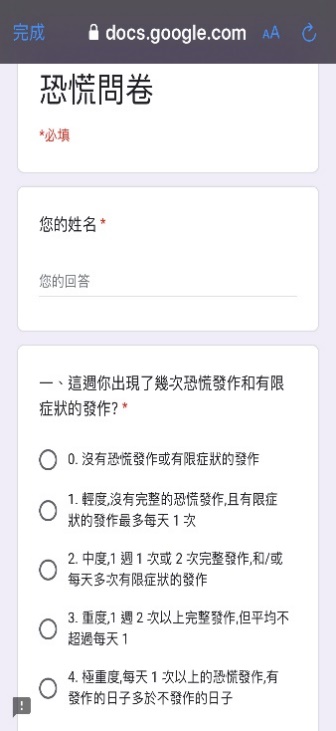

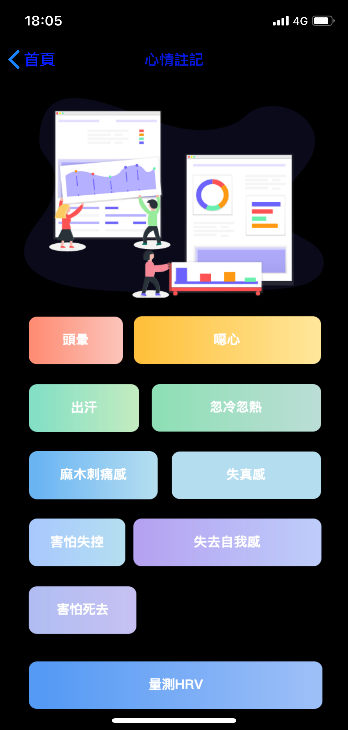

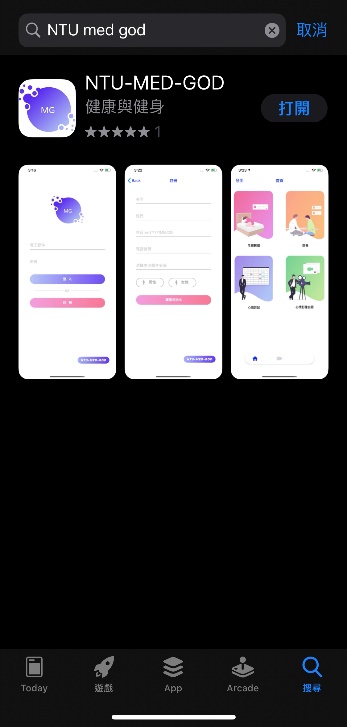

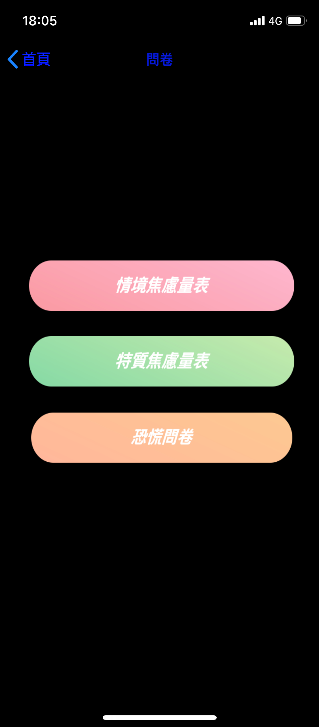


Appendix Figure 3

Part 3 Panic Monitoring Platform for Case Managers and Clinicians


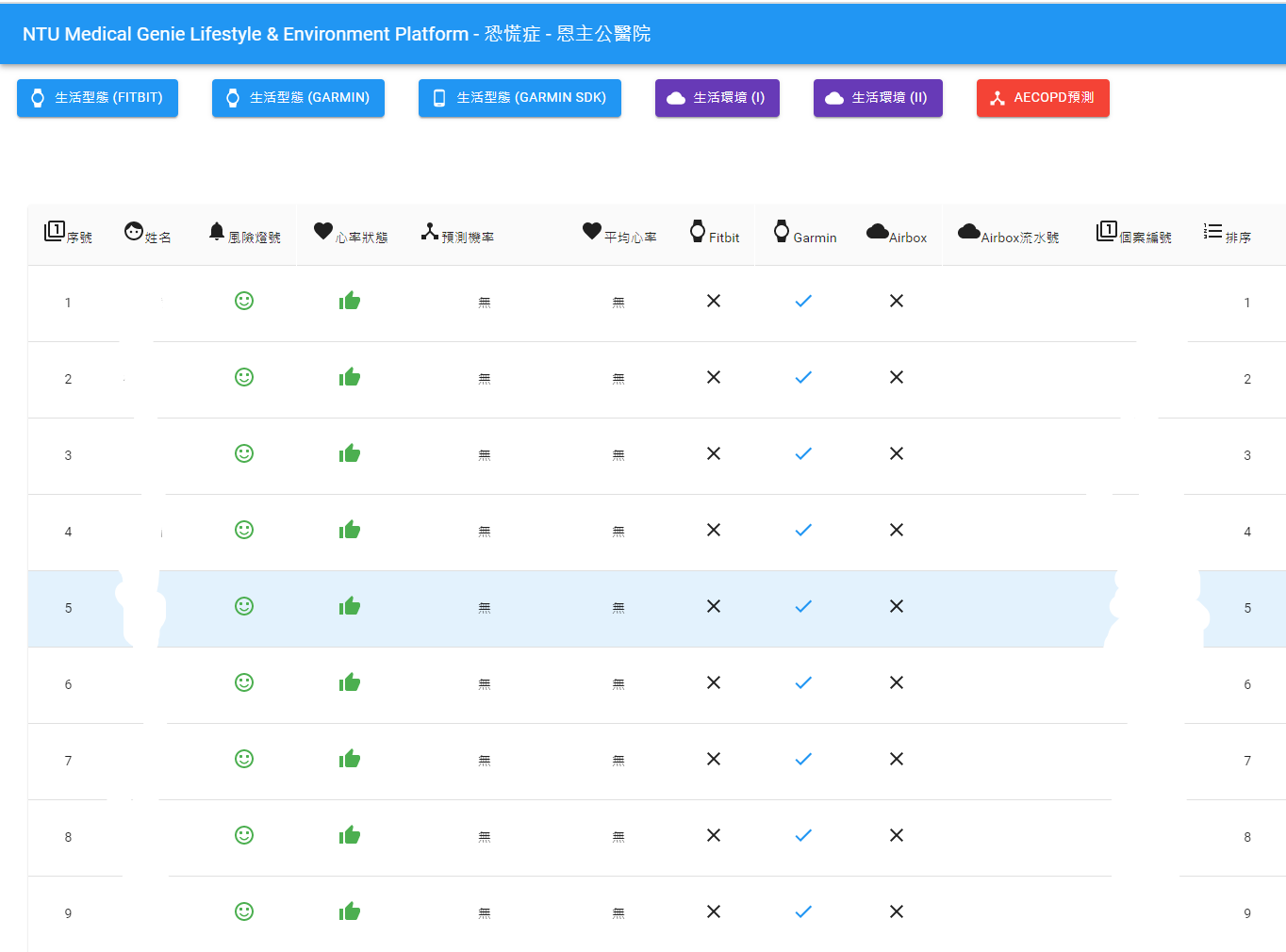


Part 4 Qualitative Assessment

- 1. We applied open-ended questions initially for the purpose of flexibility and genuine response from the participants:

1. Can you describe your experiences with the treatment?
2. How has the treatment affected your panic symptoms and avoidance behaviors?
3. Can you discuss your satisfaction with the interpersonal support you received?
4. Can you describe any visits to the emergency department due to panic attacks during the treatment period?
5. How has the treatment affected your self-recognition and management of panic symptoms?
6. Can you discuss your motivation to adhere to and maintain the treatment regimen?
   1. Semi-opened questions were applied to collect consistent data at the next stop.
7. Remission status:

"Can you describe your experience when you were considered in remission? How long did this period last, and what did it feel like not having a panic attack or worrying significantly about having one?"

1. Emergency department visits:

"Have you visited the emergency department due to panic attacks during your treatment period? If so, could you discuss your experiences, symptoms, and feelings during these visits?"

1. Interpersonal support:

"Could you discuss the kind of support you received from your case manager? How has their support through face-to-face or telephone interviews impacted your recovery process?"

1. Reassurance and self-recognition:

"How has regular case management affected your ability to manage panic symptoms? Can you talk about any changes in your self-recognition and understanding of your panic symptoms?"

1. Care coordination:

"Can you describe your experiences with care coordination during your treatment? How has the coordination between your case manager and other medical professionals affected your treatment journey?"

1. Medical adherence:

"How has your understanding of your panic disorder and the purpose of your treatment plan influenced your motivation to adhere to it? Can you discuss your feelings about continuing the maintenance phase of treatment even after being remitted from panic symptoms?"

4.2 For less talkative patients, we allowed flexibility in exploring participants' experiences while covering critical areas of interest with

4-3 Text analysis process:
Our study analyzed 46 interviews with panic disorder patients, transcribing audio files into text with Google's Cloud Speech-to-Text API and cleaning data with Python's NLTK. We performed topic modeling using the gensim library, identifying five critical themes related to mobile-aided case management like, "Interpersonal support," "Coordination," "Reassurance," and "Self-cognition," etc. We built a node structure representing these themes by importing the cleaned documents into Nvivo for further qualitative analysis. Most patients improved in "Interpersonal support" and "Self-cognition" after using mobile-aided case management. Moreover, "Coordination" emerged as a popular feature, providing a stress-free consultation process for the participants.

Part 5 Code for RMANCOVA analysis and Bonferroni adjustment by R language

R version:

Environment: R studio

library(readxl)

library(emmeans)

TAU <- read_excel("C:/Users/user/Desktop/TAU.xlsx")

MCM <- read_excel("C:/Users/user/Desktop/MCM.xlsx")

TAU$Group <- "TAU"

MCM$Group <- "MCM"

data <- rbind(TAU, MCM)

covariates <- c("Gender", "Age", "GAD", "Depression", "AG")

outcomes <- c("`PDSS-3`", "`PDSS-6`", "`PDSS-12`", "`STAIS-3`", "`STAIS-6`", "`STAIT-3`", "`STAIT-6`", "`BAI-3`", "`BAI-6`", "`BDI-3`", "`BDI-6`")

adjusted_means <- list()

for (outcome in outcomes) {

formula <- as.formula(paste(outcome, "~ Group +", paste(covariates, collapse = " + ")))

ancova_result <- aov(formula, data = data)

emm_result <- emmeans(ancova_result, "Group")

adjusted_means[[outcome]] <- summary(emm_result)$emmean

}

adjusted_means

pairwise_results <- list()

for (outcome in outcomes) {

formula <- as.formula(paste(outcome, "~ Group +", paste(covariates, collapse = " + ")))

ancova_result <- aov(formula, data = data)

emm_result <- emmeans(ancova_result, "Group")

pairwise_result <- pairs(emm_result)

pairwise_results[[outcome]] <- summary(pairwise_result, adjust = "bonferroni")$p.value

}

pairwise_results

> adjusted_means

$``PDSS-3``

[1] -5.298083 -4.981650

$``PDSS-6``

[1] -7.544450 -5.867917

$``PDSS-12``

[1] -6.629125 -5.514836

$``STAIS-3``

[1] -11.990118 -9.745794

$``STAIS-6``

[1] -17.40871 -15.64337

$``STAIT-3``

[1] -11.82498 -12.53154

$``STAIT-6``

[1] -7.241428 -7.983495

$``BAI-3``

[1] -8.878898 -6.611915

$``BAI-6``

[1] -12.755790 -4.832087

$``BDI-3``

[1] -3.037941 -1.954349

$``BDI-6``

[1] -5.369511 -4.224342

> pairwise_results <- list()

>

> for (outcome in outcomes) {

+ formula <- as.formula(paste(outcome, "~ Group +", paste(covariates, collapse = " + ")))

+ ancova_result <- aov(formula, data = data)

+ emm_result <- emmeans(ancova_result, "Group")

+ pairwise_result <- pairs(emm_result)

+ pairwise_results[[outcome]] <- summary(pairwise_result, adjust = "bonferroni")$p.value

+ }

>

> pairwise_results

$``PDSS-3``

[1] 0.4936502

$``PDSS-6``

[1] 0.007663783

$``PDSS-12``

[1] 0.03501357

$``STAIS-3``

[1] 0.02791377

$``STAIS-6``

[1] 0.01580412

$``STAIT-3``

[1] 0.3249492

$``STAIT-6``

[1] 0.3975468

$``BAI-3``

[1] 0.00018466

$``BAI-6``

[1] 1.422751e-16

$``BDI-3``

[1] 0.02704301

$``BDI-6``

[1] 0.001392242

> confidence_intervals <- list()

>

> for (outcome in outcomes) {

+ formula <- as.formula(paste(outcome, "~ Group +", paste(covariates, collapse = " + ")))

+ ancova_result <- aov(formula, data = data)

+ emm_result <- emmeans(ancova_result, "Group")

+ pairwise_result <- pairs(emm_result)

+ confidence_intervals[[outcome]] <- confint(pairwise_result, adjust = "bonferroni")

+ }

> > confidence_intervals

$``PDSS-3``

contrast estimate SE df lower.CL upper.CL

MCM - TAU -0.316 0.461 131 -1.23 0.595

Results are averaged over the levels of: Gender, GAD, Depression, AG

Confidence level used: 0.95

$``PDSS-6``

contrast estimate SE df lower.CL upper.CL

MCM - TAU -1.68 0.619 131 -2.9 -0.452

Results are averaged over the levels of: Gender, GAD, Depression, AG

Confidence level used: 0.95

$``PDSS-12``

contrast estimate SE df lower.CL upper.CL

MCM - TAU -1.11 0.523 131 -2.15 -0.0796

Results are averaged over the levels of: Gender, GAD, Depression, AG

Confidence level used: 0.95

$``STAIS-3``

contrast estimate SE df lower.CL upper.CL

MCM - TAU -2.24 1.01 131 -4.24 -0.247

Results are averaged over the levels of: Gender, GAD, Depression, AG

Confidence level used: 0.95

$``STAIS-6``

contrast estimate SE df lower.CL upper.CL

MCM - TAU -1.77 0.722 131 -3.19 -0.337

Results are averaged over the levels of: Gender, GAD, Depression, AG

Confidence level used: 0.95

$``STAIT-3``

contrast estimate SE df lower.CL upper.CL

MCM - TAU 0.707 0.715 131 -0.708 2.12

Results are averaged over the levels of: Gender, GAD, Depression, AG

Confidence level used: 0.95

$``STAIT-6``

contrast estimate SE df lower.CL upper.CL

MCM - TAU 0.742 0.874 131 -0.987 2.47

Results are averaged over the levels of: Gender, GAD, Depression, AG

Confidence level used: 0.95

$``BAI-3``

contrast estimate SE df lower.CL upper.CL

MCM - TAU -2.27 0.589 131 -3.43 -1.1

Results are averaged over the levels of: Gender, GAD, Depression, AG

Confidence level used: 0.95

$``BAI-6``

contrast estimate SE df lower.CL upper.CL

MCM - TAU -7.92 0.835 131 -9.58 -6.27

Results are averaged over the levels of: Gender, GAD, Depression, AG

Confidence level used: 0.95

$``BDI-3``

contrast estimate SE df lower.CL upper.CL

MCM - TAU -1.08 0.485 131 -2.04 -0.125

Results are averaged over the levels of: Gender, GAD, Depression, AG

Confidence level used: 0.95

$``BDI-6``

contrast estimate SE df lower.CL upper.CL

MCM - TAU -1.15 0.351 131 -1.84 -0.452

Results are averaged over the levels of: Gender, GAD, Depression, AG

Confidence level used: 0.95

For Baseline:

> library(readxl)

> library(emmeans)

>

> TAU_base <- read_excel("C:/Users/user/Desktop/TAU_base.xlsx")

> MCM_base <- read_excel("C:/Users/user/Desktop/MCM_base.xlsx")

>

> TAU_base$Group <- "TAU_base"

> MCM_base$Group <- "MCM_base"

> data <- rbind(TAU_base, MCM_base)

>

> covariates <- c("Gender", "Age", "GAD", "Depression", "AG")

> outcomes <- c("`PDSS-0`", "`STAIS-0`", "`STAIT-0`", "`BAI-0`", "`BDI-0`")

>

> adjusted_means <- list()

>

> for (outcome in outcomes) {

+ formula <- as.formula(paste(outcome, "~ Group +", paste(covariates, collapse = " + ")))

+ ancova_result <- aov(formula, data = data)

+ emm_result <- emmeans(ancova_result, "Group")

+ adjusted_means[[outcome]] <- summary(emm_result)$emmean

+ }

>

> print(adjusted_means)

$``PDSS-0``

[1] 9.521779 11.492359

$``STAIS-0``

[1] 44.98803 45.48798

$``STAIT-0``

[1] 46.09251 42.60942

$``BAI-0``

[1] 23.63911 21.00118

$``BDI-0``

[1] 16.46394 19.74199

> pairwise_results <- list()

>

> for (outcome in outcomes) {

+ formula <- as.formula(paste(outcome, "~ Group +", paste(covariates, collapse = " + ")))

+ ancova_result <- aov(formula, data = data)

+ emm_result <- emmeans(ancova_result, "Group")

+ pairwise_result <- pairs(emm_result)

+ pairwise_results[[outcome]] <- summary(pairwise_result, adjust = "bonferroni")$p.value

+ }

>

> # Bonferroni adjusted p-value

> print(pairwise_results)

$``PDSS-0``

[1] 0.05055312

$``STAIS-0``

[1] 0.8108526

$``STAIT-0``

[1] 0.04384956

$``BAI-0``

[1] 0.2913924

$``BDI-0``

[1] 0.1380756

> confidence_intervals <- list()

> # Compute confidence intervals for the difference between adjusted group means

> for (outcome in outcomes) {

+ formula <- as.formula(paste(outcome, "~ Group +", paste(covariates, collapse = " + ")))

+ ancova_result <- aov(formula, data = data)

+ emm_result <- emmeans(ancova_result, "Group")

+ pairwise_result <- pairs(emm_result)

+ confidence_intervals[[outcome]] <- confint(pairwise_result)

+ }

>

> # Print the confidence intervals

> print(confidence_intervals)

$``PDSS-0``

contrast estimate SE df lower.CL upper.CL

MCM_base - TAU_base -1.97 0.999 131 -3.95 0.00482

Results are averaged over the levels of: Gender, GAD, Depression, AG

Confidence level used: 0.95

$``STAIS-0``

contrast estimate SE df lower.CL upper.CL

MCM_base - TAU_base -0.5 2.08 131 -4.62 3.62

Results are averaged over the levels of: Gender, GAD, Depression, AG

Confidence level used: 0.95

$``STAIT-0``

contrast estimate SE df lower.CL upper.CL

MCM_base - TAU_base 3.48 1.71 131 0.0975 6.87

Results are averaged over the levels of: Gender, GAD, Depression, AG

Confidence level used: 0.95

$``BAI-0``

contrast estimate SE df lower.CL upper.CL

MCM_base - TAU_base 2.64 2.49 131 -2.29 7.56

Results are averaged over the levels of: Gender, GAD, Depression, AG

Confidence level used: 0.95

$``BDI-0``

contrast estimate SE df lower.CL upper.CL

MCM_base - TAU_base -3.28 2.2 131 -7.62 1.07

Results are averaged over the levels of: Gender, GAD, Depression, AG

Confidence level used: 0.95

> # Compute and print adjusted group means and their standard deviations

> adjusted_means <- list()

> adjusted_sd <- list()

>

> for (outcome in outcomes) {

+ formula <- as.formula(paste(outcome, "~ Group +", paste(covariates, collapse = " + ")))

+ ancova_result <- aov(formula, data = data)

+ emm_result <- emmeans(ancova_result, "Group")

+ adjusted_means[[outcome]] <- summary(emm_result)$emmean

+ adjusted_sd[[outcome]] <- summary(emm_result)$SE

+ }

>

> # Print adjusted group means and their standard deviations

> print(adjusted_means)

$``PDSS-0``

[1] 9.521779 11.492359

$``STAIS-0``

[1] 44.98803 45.48798

$``STAIT-0``

[1] 46.09251 42.60942

$``BAI-0``

[1] 23.63911 21.00118

$``BDI-0``

[1] 16.46394 19.74199

> print(adjusted_sd)

$``PDSS-0``

[1] 0.8095274 0.9678494

$``STAIS-0``

[1] 1.690123 2.020665

$``STAIT-0``

[1] 1.387449 1.658797

$``BAI-0``

[1] 2.018743 2.413555

$``BDI-0``

[1] 1.781031 2.129353

PART 6 Baseline comparison (non-adjusted) code using T test

> library(readxl)

>

> TAU <- read_excel("C:/Users/user/Desktop/TAU_base.xlsx")

> MCM <- read_excel("C:/Users/user/Desktop/MCM_base.xlsx")

>

> TAU$Group <- "TAU"

> MCM$Group <- "MCM"

>

> combined_data <- rbind(TAU, MCM)

>

> variables <- c("PDSS-0", "STAIS-0", "STAIT-0", "BAI-0", "BDI-0")

>

> for(var in variables) {

+ t_test_result <- t.test(combined_data[combined_data$Group == "TAU", var],

+ combined_data[combined_data$Group == "MCM", var])

+ cat(paste("p-value for", var, ":", round(t_test_result$p.value, 4), "\n"))

+ }

p-value for PDSS-0 : 0.0026

p-value for STAIS-0 : 0.8895

p-value for STAIT-0 : 0.0203

p-value for BAI-0 : 0.2481

p-value for BDI-0 : 0.031

PART 7 Levene’s Test code for variance examination between disparity in sample sizes (n=108 vs. n=30)

> library(car)

>

> for(var in covariates_subset) {

+ print(paste("Levene's Test for", var))

+ print(leveneTest(combined_data[, var], combined_data$Group))

+ }

[1] "Levene's Test for Gender"

Levene's Test for Homogeneity of Variance (center = median)

Df F value Pr(>F)

group 1 0.0264 0.8712

136

[1] "Levene's Test for Age"

Levene's Test for Homogeneity of Variance (center = median)

Df F value Pr(>F)

group 1 3.4668 0.06477 .

136

---

Signif. codes: 0 ‘***’ 0.001 ‘**’ 0.01 ‘*’ 0.05 ‘.’ 0.1 ‘ ’ 1

[1] "Levene's Test for GAD"

Levene's Test for Homogeneity of Variance (center = median)

Df F value Pr(>F)

group 1 3.8861 0.05072 .

136

---

Signif. codes: 0 ‘***’ 0.001 ‘**’ 0.01 ‘*’ 0.05 ‘.’ 0.1 ‘ ’ 1

[1] "Levene's Test for Depression"

Levene's Test for Homogeneity of Variance (center = median)

Df F value Pr(>F)

group 1 7.6117 0.006598 **

136

---

Signif. codes: 0 ‘***’ 0.001 ‘**’ 0.01 ‘*’ 0.05 ‘.’ 0.1 ‘ ’ 1

[1] "Levene's Test for AG"

Levene's Test for Homogeneity of Variance (center = median)

Df F value Pr(>F)

group 1 0.6731 0.4134

136
